# Posttraumatic Stress Symptoms and Pain Sensitization After Whiplash Injury: A Longitudinal Cohort Study With Quantitative Sensory Testing

**DOI:** 10.3389/fpain.2022.908048

**Published:** 2022-06-15

**Authors:** Tonny Elmose Andersen, Sophie Lykkegaard Ravn, Tina Carstensen, Eva Ørnbøl, Lisbeth Frostholm, Helge Kasch

**Affiliations:** ^1^Department of Psychology, University of Southern Denmark, Odense, Denmark; ^2^Specialized Hospital for Polio and Accident Victims, Roedovre, Denmark; ^3^The Research Department of Functional Disorders and Psychosomatics, Aarhus University Hospital, Aarhus, Denmark; ^4^Department of Clinical Medicine, Health, Aarhus University, Aarhus, Denmark; ^5^Department of Neurology, Viborg Regional Hospital, Viborg, Denmark

**Keywords:** posttraumatic stress, whiplash, quantitative sensory tests, QST, sensitization, pain

## Abstract

Posttraumatic stress symptoms (PTSS) are common after whiplash injury and are associated with poor recovery. The acute stress response may lead to pain sensitization and widespread pain, thereby compromising recovery. To our knowledge, no longitudinal study has assessed the associations between early PTSS and pain sensitization over time using quantitative sensory testing (QST). The aim of this study was to compare participants with different levels of PTSS, as measured by the impact of event scale (IES; subclinical 0–8, mild 9–25, and clinical ≥ 26) at baseline (<10-day post-injury) and at a follow-up of 1, 3, 6, and 12-month post-injury on pain sensitivity, neck mobility, pain distribution, and pain intensity. In total, 740 participants were recruited from emergency units or general practitioners with acute neck pain after a whiplash injury. The clinical PTSS group showed increased pain sensitivity on all QSTs at all time points compared to the subclinical PTSS group. Also, the clinical PTSS group showed significantly lower neck mobility at all time points except for a 3-month follow-up compared to the subclinical PTSS group. Moreover, the clinical PTSS group showed more widespread pain and self-reported headache and neck pain intensity at all time points compared to the subclinical PTSS group. This study emphasizes that participants with clinical levels of PTSS constitute a high-risk group that is sensitized to pain early after the injury. Hence, screening for PTSS within the 1st week after whiplash injury for those who experience high levels of pain intensity and distress may be an important clinical procedure in the assessment and treatment of whiplash-associated disorders (WAD).

## Introduction

Long-term posttraumatic pain is the second most prevalent pain condition after musculoskeletal pain (1). In particular, whiplash injury is a major contributor to long-term pain and disability after a motor vehicle collision (MVC) (2). A previous systematic review concluded that up to 50% of participants continue to report symptoms 1-year post-injury (3), and more recent trajectory studies show that about one in five participants continue to have severe symptoms over time (4, 5). Elevated levels of posttraumatic stress symptoms (PTSS) are common in whiplash and are associated with poor recovery and higher pain levels after a whiplash injury (4, 6, 7). Acute stress response mechanisms in the early posttrauma phase may lead to sensitization and pain and result in hyperalgesia due to the activation of the hypothalamic-pituitary-adrenal (HPA) system (8). After a whiplash injury, such mechanisms have further been associated with the development of muscle degeneration and observed restricted active cervical range of movement [for an overview, see (9)]. While these findings might be due to disuse, the level of posttraumatic traumatic stress reactions and not the reduced range of motion (ROM) after whiplash injury was found to mediate the relationship between acute pain and fatty infiltrates in the neck muscles 6-month post-injury (10). Hence, posttraumatic stress reactions may result in both peripheral and central sensitization and changes in muscle morphology (11).

Few studies have assessed the underlying mechanisms of pain in PTSS and often with opposite findings of either increased or decreased sensitivity to pain or unaltered pain processing (12). In a recent meta-analysis assessing experimentally evoked pain perception in participants with high levels of PTSS, no main effect of PTSS was found (12). However, stratification according to trauma type showed that accident-related PTSS, i.e., after a whiplash injury, was associated with increased sensitivity to pain (12), indicating the existence of different subgroups with qualitative differences in pain processing.

Largely, there is a consensus that ongoing symptoms after whiplash injury—collectively known as whiplash-associated disorders (WAD)—are associated with an altered sensory processing pattern characterized by decreased pressure pain detection thresholds (PPDTs) both close to the injury site and remotely, as signs of both peripheral and central sensitization to pain (13). Central sensitization, expressed as increased sensitivity to pressure pain at remote sites in uninjured tissue, is often associated with poorer recovery after a whiplash injury (9). While decreased PPDT has also been reported in idiopathic neck pain, widespread hypersensitivity to pressure pain is only found in WAD (14). Widespread pain is commonly encountered after MVC, with more than 20% reporting widespread pain (15). When tested in the neck, PPDT values <210 kPa for men and <185 kPa for women are considered below the normative compared to uninjured controls (16, 17). Finally, participants with WAD have a reduced cervical ROM as compared to participants with idiopathic neck pain (18).

Taken together, the abovementioned findings indicate that the posttraumatic nature of whiplash injury may pose a substantial risk of sensitization to pain and non-recovery. Unfortunately, the majority of studies addressing the association between PTSS and pain sensitization are either cross-sectional or based solely on self-report questionnaires. To our knowledge, no longitudinal study has assessed associations between early PTSS and pain sensitization patterns over time using quantitative sensory testing (QST). Therefore, this was the aim of this study using data from the first 10 days after a whiplash injury and over the course of the first year post-injury. The objectives were to compare participants with different levels of PTSS (subclinical, mild, and clinical symptom levels) at baseline on pain sensitivity, neck mobility, pain intensity, and pain distribution at all time points. Explicit hypotheses were made for the differences between clinical and subclinical levels of PTSS, while the comparisons with the mild group were explorative. Specifically, it was hypothesized that:

1. Participants with clinical PTSS at baseline experience increased pain sensitivity at all time points as compared to participants with subclinical PTSS.
2. Participants with clinical PTSS at baseline experience restricted neck mobility at all time points as compared to participants with subclinical PTSS.
3. Participants with clinical PTSS at baseline experience increased pain distribution at all time points as compared to participants with subclinical PTSS.
4. Participants with clinical PTSS at baseline report increased pain intensity at all time points as compared to participants with subclinical PTSS.

## Materials and Methods

### Design and Sample

This study is a prospective, multicenter cohort study. The current study consists of secondary analyses performed on the entire cohort. Within the framework of this study, two randomized controlled trials (RCTs) were performed. The RCTs included only a subgroup of the overall sample. For a description of the RCT studies, please see the work of Kongsted et al. (19, 20).

Participants consulting emergency units or general practitioners with acute neck pain after rear-end or side-impact car collision were invited to participate in this multicenter study conducted by the Danish Pain Research Center, Aarhus University Hospital, Aarhus, Denmark, and the Back Research Center, Odense University Hospital, Ringe, Denmark. The uptake area covered 1.7 million inhabitants in 2001. Participants were included in the study from April 2001 to June 2003.

Participants were informed about the study by written invitation. Inclusion criteria were participants aged between 18 and 70 years experiencing neck pain within 72 h after being exposed to a rear-end or side-impact car collision. Exclusion criteria were as follows: participants could not be examined within 10 days of the car accident, fractures, or dislocations of the cervical spine, retrograde or anterograde amnesia or unconsciousness in relation to the accident, injuries other than the whiplash trauma, no symptoms, significant pre-collision physical or psychiatric disorder, self-reported average neck pain during the preceding 6 months exceeding five on a box scale 0–10, and alcohol or drug abuse. All eligible and interested participants were included within the first 10 days after the collision, where they completed the baseline questionnaire, clinical examinations, and QSTs. In addition, data were collected at 1, 3, 6, and 12 months post-inclusion with different data for the different time points, which are outlined below.

### Measures

Data used in this study consist of a combination of data from self-reported questionnaires, clinical examinations, and QSTs. Questionnaires were used at baseline and at 3, 6, and 12 months post-injury, while clinical examinations and QSTs were conducted at baseline and at a 1-, 3-, and 12-month follow-up. The examinations included a neurological examination and measurements of neck mobility and pain tests. The neurological examination was performed according to common clinical standards and included cranial nerve function, muscle strength, tonus, tendon reflexes, and sensory testing. These different measures and their purpose are described below in detail in this study.

#### Posttraumatic Stress Symptoms

Acute levels of PTSS were measured at baseline within 10 days after injury using the impact of event scale (IES) questionnaire (21). The scale was developed to measure subjective distress related to a specific event, and, while it did not correspond to PTSD as defined by a diagnostic system, it could be used to indicate PTSS. The scale is a 15-item self-report questionnaire that assesses experiences of avoidance and intrusion during the last week on a four-point Likert scale (0 = not at all, 1 = rarely, 3 = sometimes, and 5 = often). The total score ranges from 0 to 75, with higher scores indicating more severe PTSS. Based on clinical cut-off criteria (21, 22), three PTSS groups were defined for this study as follows: subclinical (0–8), mild (9–25), and clinical (≥26).

#### Pain Sensitivity Using QSTs

Two indicators of pain sensitivity using QSTs were used in this study: PPDT and pressure pain tolerance (PPT). Pressure algometry was performed with the Somedic Type 1 algometer (Solentuna, Sweden) using both PPDT (in triplets) and PPT thresholds (single measures). They were measured at baseline and 1, 3, and 12 months post-injury. To examine deep PPDTs, participants were instructed to push a button when the sensation changed from one of pressure alone to one of both pressure and pain (23, 24). The procedure was performed at a total of 10 neck and jaw muscle spots (the temporal muscle (L/R), the masseter muscle (L/R), the sternocleidomastoid at the proximal insertion (L/R), the trapezius muscle at the superior portion (L/R), the infraspinatus (L/R), and at the two control sites in the upper extremity at the third left interphalangeal joint and the lower extremity at the anterior tibal muscle (left). The probe was applied at an intended angle of 90° to the examined area, and the slope was set to 30 kPa/s, with a standard probe of 1 cm^2^. To examine the PPT of deep tissue, participants were instructed to press the button, not at the time point when experiencing pain, but at the point of change when the pain experience was not tolerable. Pressure pain change per second was similar during the PPT and PPDT examinations and rose to the slope of 30 kPa/s. PPT was examined at the left masseter muscle, the infraspinatus muscle, and at the two control sites situated in the upper extremity at the third left interphalangeal joint and the lower extremity at the anterior tibal muscle (left) (25). Mean scores for PPT and PPDT were used as outcomes.

#### Neck Mobility

According to previous descriptions (26), active neck mobility was measured as the maximum active ROM in three dimensions by mounting a cervical range of motion (CROM) device (Performance Attainment Associates, Roseville, MN) with two goniometers (measuring flexion-extension and lateral flexion) and a magnetic plane meter. During measurement, the following ones were registered: (1) active neck flexion (during jaw retraction), (2) active neck extension, (3) right lateroflexion, (4) left lateroflexion, (5) right rotation, and (6) left rotation (following a horizontal line from the neutral position). The individual scores (degrees of movement) were registered, and the total sum of all six directions (TotalCROM) was computed. Furthermore, the presence (y/n) of local and distant pain during each of the six movements was recorded (measuring the rotation). Neck mobility was examined at baseline and 1, 3, and 12 months post-injury.

#### Pain Distribution

Two indicators of pain distribution were used: self-reported number of body areas with pain and a clinical examination of palpation.

The number of body areas with pain was measured as the total number of painful areas marked on the McGill Pain Map (27). The body map was filled out at baseline and after 3 and 12 months.

Methodic palpation was performed at baseline and after 1, 3, and 12 months using a previously developed scheme (25). Quantification of tenderness by palpation and the use of pressure algometers as an indicator for pain distribution (23). Palpation was performed at nine sites bilaterally: (1) the anterior and (2) posterior part of the temporal muscle, (3) the masseter muscle at the mandibular angle, (4) the lateral pterygoid muscle, (5) the sternocleid at the insertion point, (6) the sternocleidomastoid muscle at its belly, (7) the suboccipital muscle, (8) the trapezius at its superior part, and (9) the rhomboid muscle situated at the medial border of the scapula. A standard pressure using fingers two and three with slightly rotating fingertips was applied at firm locations, and a three-finger grip was applied over soft anatomic structures. A score of 0 to 4 points was given at each site according to the American College of Rheumatology (ACR) criteria: 0 = no pain (denial of tenderness), 1 = mild pain (complaint of pain without a grimace, flinch, or withdrawal), 2 = moderate pain (complaint of pain plus grimace or flinch), 3 = severe pain (complaint of pain plus marked flinch or withdrawal), and 4 = unbearable pain (participant is untouchable, withdraws without palpation) (28). The sum score for all sites was used as an outcome.

#### Pain Intensity

Two indicators of pain intensity were used: neck pain intensity and the intensity of headache. Both were measured as the average level of pain during the last week on two separate 11-point box scales (0 = no pain, 10 = worst possible pain) (29). Both were reported at baseline and after 3, 6, and 12 months using questionnaires.

#### Socio-Demographics and the Use of Analgesics

The socio-demographic variables at the time of the accident included age, gender, education (“basic school” and “further education”), vocational training (“unskilled,” “skilled,” “formal education ≤4 years,” “formal education >4 years,” and “other”), work status (“student,” “self-employed,” “white collar,” “blue collar,” and “unemployed”), and living conditions (“alone,” “with partner,” “with parents,” and “other”).

Self-reported use of analgesics was recorded as yes/no answers in relation to the use of “over-the-counter drugs/week opioids” and “strong opioids” (prescription).

### Statistical Analysis

Crude comparisons between the IES score groups on categorical variables were analyzed using χ^2^. Differences between the IES score groups for continuous variables (age) were analyzed using the Kruskal–Wallis test. The statistical program used for the analysis was STATA 17.0 for Windows.

#### Multilevel Mixed-Effects Linear Models

The hypotheses were assessed by multilevel linear mixed-effects models (LMMs). These models make it possible to deal efficiently with missing values due to dropout, assuming that the dropout mechanism is missing at random (MAR). Thus, all available data were used.

Unadjusted LMMs with random intercept were used to describe the development over time of all outcomes. Each model included two explanatory variables: time of measurement (categorical) and partitioning of the PTSS score at baseline (subclinical vs. mild vs. clinical) and their interaction. The subclinical PTSS group was chosen as the reference group in the models as it was characterized by having no PTSS symptoms or symptom levels that are considered below clinical importance.

In all models, we first tested whether there were different developments over time (i.e., all interaction terms equal to 0). Next, the model estimated means were calculated for the three PTSS groups at all times of measurement and pairwise differences between PTSS groups, again at all times of measurement. Also, all models were checked by graphical inspection of the distribution of the residuals and random intercepts.

## Results

A total of 1,495 participants were assessed for eligibility. Among these, 548 were ineligible, 200 declined, and seven were excluded due to protocol violations. A large part (22.6%) of the ineligible participants could not be examined within 10 days after the collision, and 17.7% had injuries other than whiplash trauma, leaving a total of 740 included participants. Data on 737 participants were included in the analyses (three participants did not have any PTSS score).

In total, 64.1% of the sample were women, and the mean age of the participants was 34.8 years of age. Further demographic characteristics and descriptive statistics are presented in Table 1. There were significantly more women in the clinical PTSS group (76.1%) compared to the subclinical group (55.8%). Finally, higher use of both mild and strong opioids was reported in the clinical PTSS group compared to the other groups. However, only the use of mild opioids was significantly different from the other PTSS groups (see Table 2 for details).

**Table 1:** Descriptive characteristics at baseline of the overall sample and the three posttraumatic stress symptoms (PTSS) groups.

			**PTSS groups**					
		**All** ***N*** **=** **740**	**Subclinical** ***N*** **=** **362**	**Mild** ***N*** **=** **283**	**Clinical** ***N*** **=** **92**	**Kruskal–Wallis test**		
						***χ*** ^**2**^	**df**	***p***
**Mean age years (SD)**		34.8 (11.4)	34.4 (11.2)	35.5 (11.4)	34.6 (12.7)	1.8	2	0.402
						**χ^2^ test**		
						χ^2^	df	*p*
**Gender (%)**	Female	64.1	55.8	70.7	76.1	21.9	2	<0.001
**Work status (%)**						18.4	15	0.241
	Self-employed	4.9	3.0	7.4	4.4			
	White collar	40.1	41.7	41.0	30.4			
	Blue collar	22.7	25.1	19.1	23.9			
	Student	20.4	19.6	19.4	27.2			
	Unemployed	10.0	8.3	11.3	13.0			
	Unaccounted	1.9	2.2	1.8	1.1			
**Living conditions (%)**						11.2	12	0.510
	With partner	69.9	73.2	66.8	66.3			
	Alone	18.4	14.9	22.2	19.6			
	With parents	8.8	9.4	7.8	9.8			
	Other	2.7	1.9	3.2	4.4			
	Unaccounted	0.3	0.6	0.0	0.0			
**Education (%)**						2.475	6	0.871
	Basic school (7^th^–10^th^)	48.7	46.4	50.5	51.1			
	Further education	50.7	52.8	49.1	47.9			
	Unaccounted	0.7	0.8	0.4	1.1			
**Vocational training (%)**						15.8	15	0.394
	Unskilled	20.3	17.7	20.5	30.4			
	Skilled	31.2	30.7	31.8	30.4			
	Formal education <4yrs	22.6	23.5	24.0	15.2			
	Formal education ≥ 4yrs	8.5	9.4	7.4	7.6			
	Other	11.2	11.9	10.2	12.0			
	Unaccounted	6.2	6.9	6.0	4.4			

*PTSS groups = Impact of event scale (IES) scores: subclinical (0–8), mild (9–25), and clinical (≥ 26)*.

**Table 2:** Use of analgesics at baseline for the overall sample and the three PTSS groups.

	**Yes**	**No**	**All**	**χ^2^ test**
Whiplash-related use of analgesics, all	572	164	736	*p* = 0.010
Subclinical	269	95	364	
Mild	222	58	280	
Clinical	81	11	92	
Use of weak opioids/OtC, all	563	170	733	*p* = 0.015
Subclinical	265	98	363	
Mild	220	60	280	
Clinical	78	12	90	
Use of strong opioids, all	39	697	736	*p* = 0.110
Subclinical	18	346	364	
Mild	12	268	280	
Clinical	9	83	92	

*PTSS groups = Impact of event scale (IES) scores: subclinical (0–8), mild (9–25), and clinical (≥ 26). OtC, Over the counter drugs*.

### PTSS Groups and Pain Sensitivity Over Time

**Figure 1:**
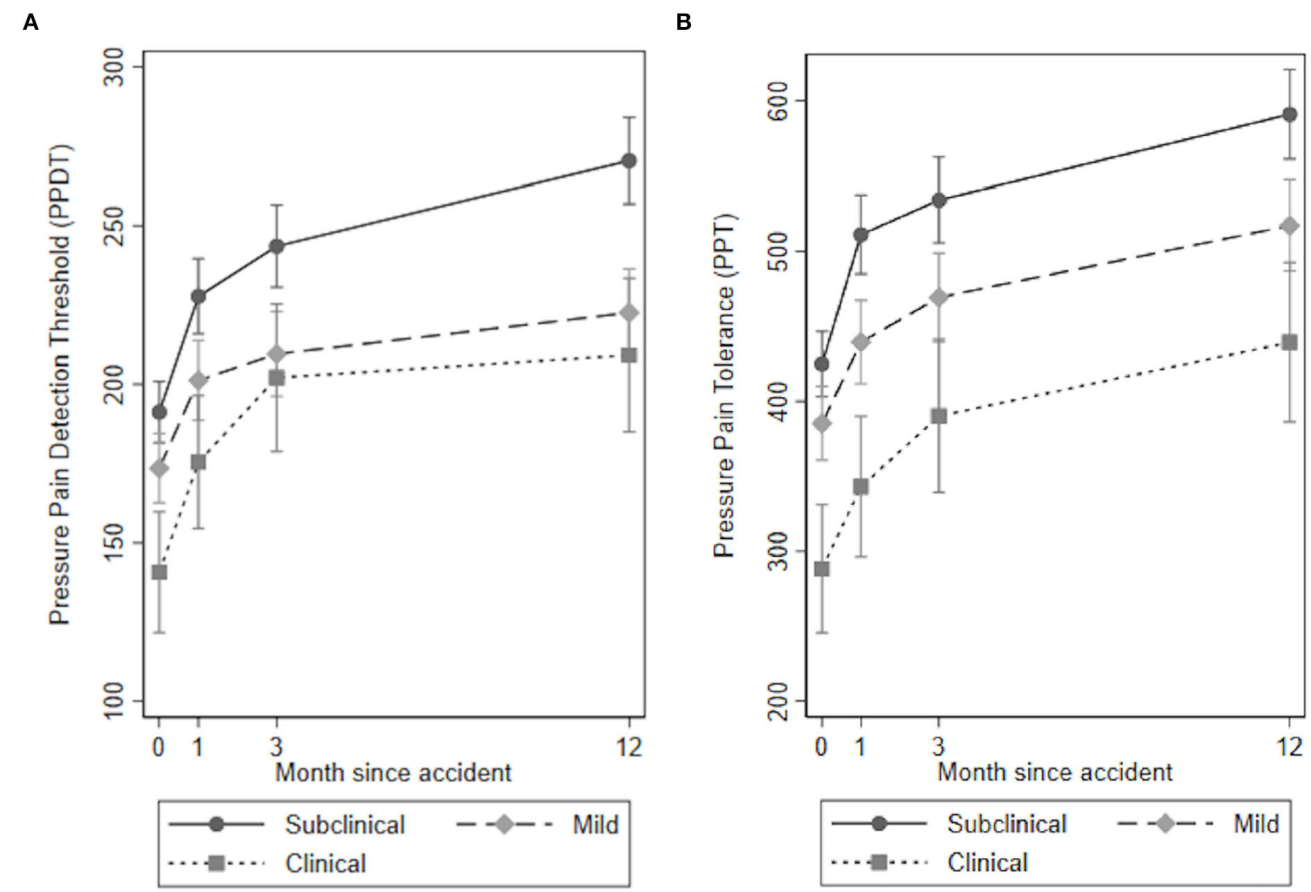
**(A,B)** Pressure pain detection threshold (PPDT) and pressure pain tolerance (PPT) by posttraumatic stress symptoms (PTSS) groups over time.

**Figure 2:**
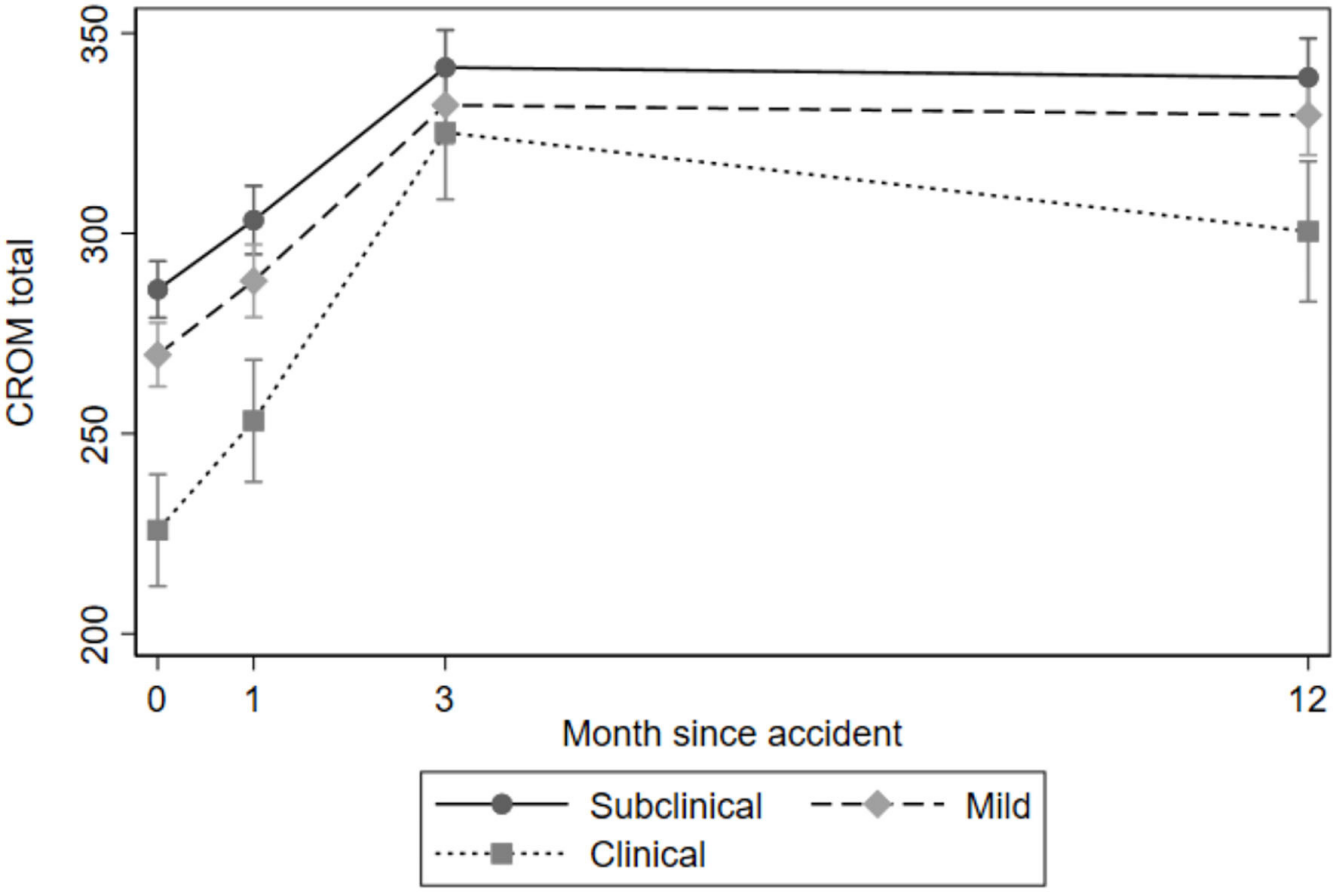
Cervical range of motion by PTSS groups over time.

**Figure 3:**
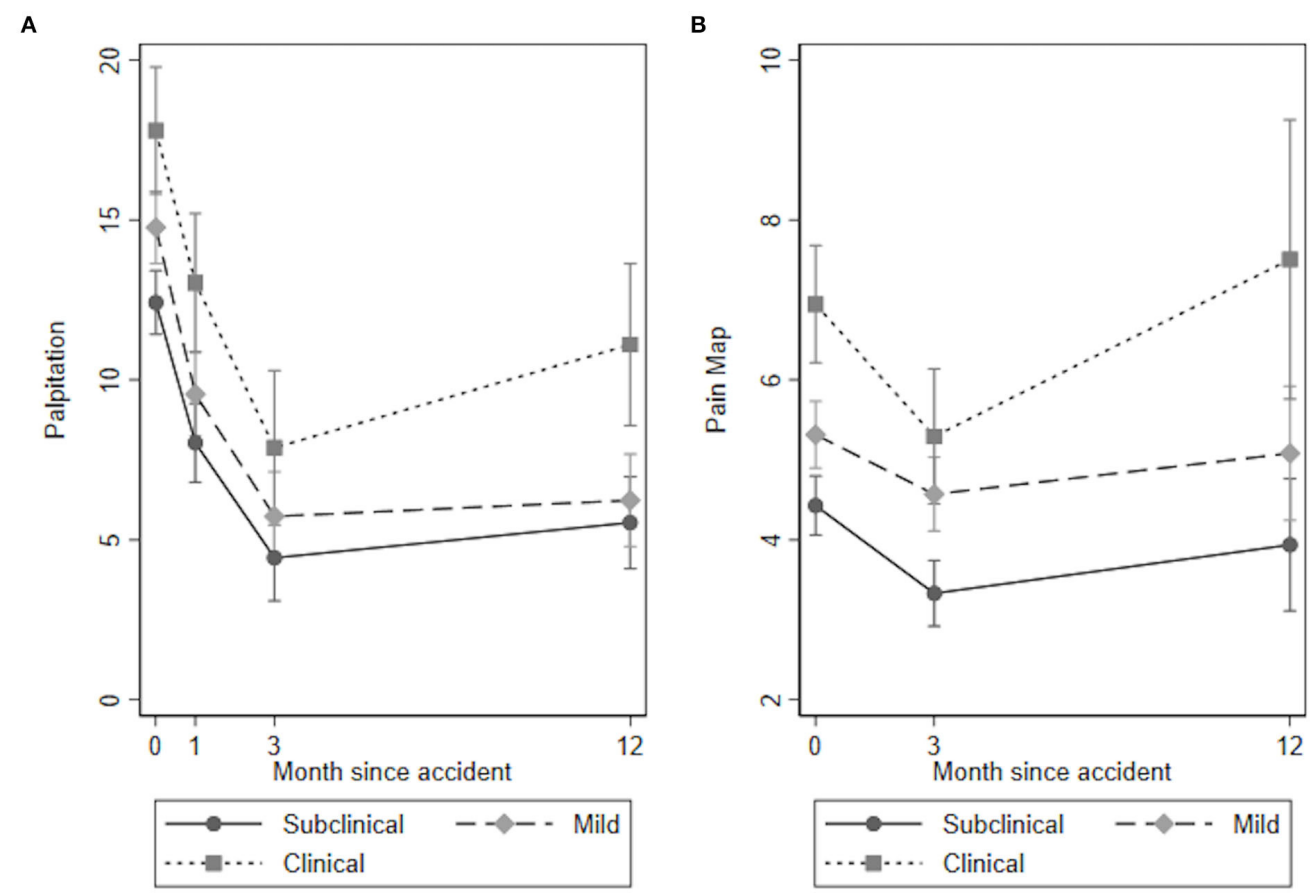
Pain Distribution: **(A)** Palpation and **(B)** Pain Map.

**Figure 4:**
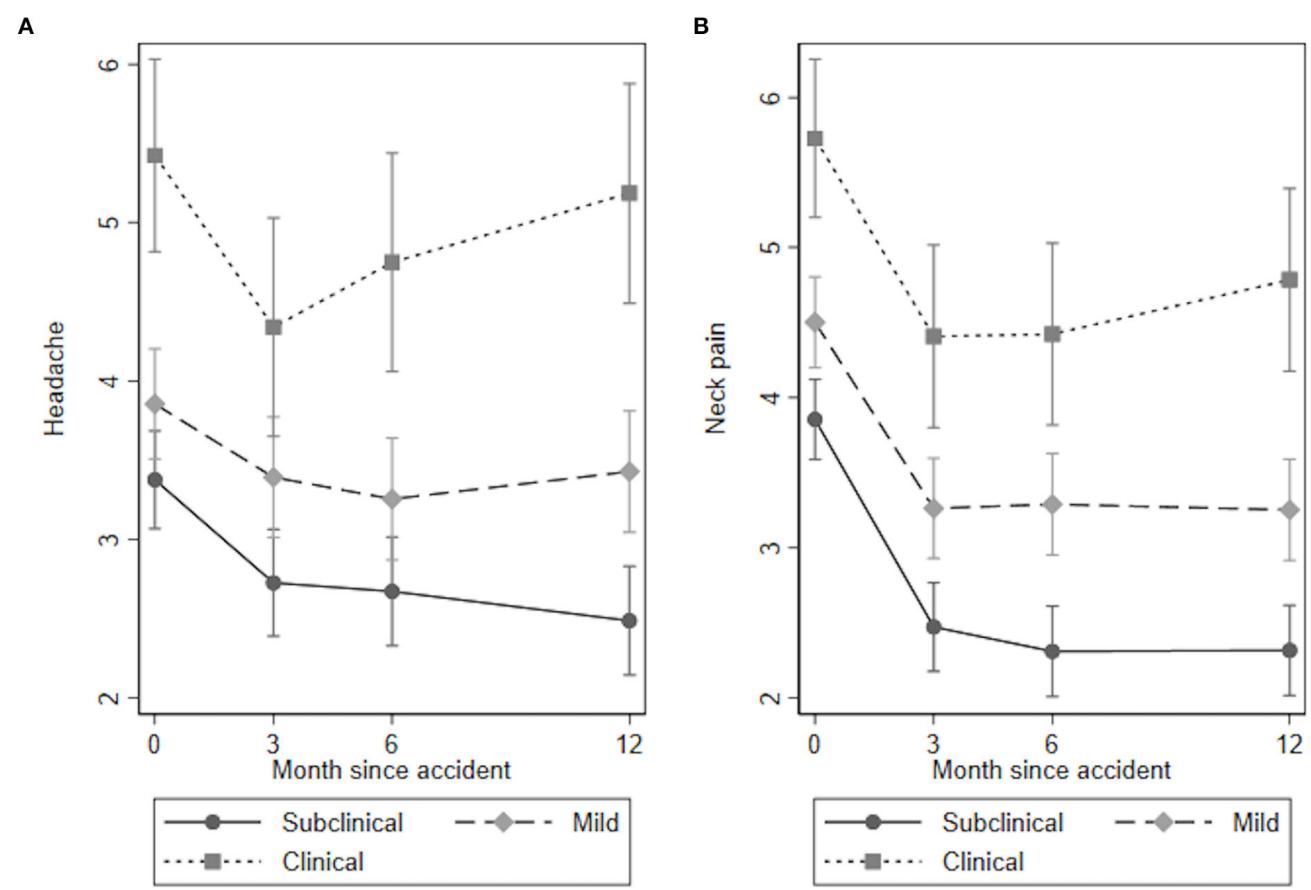
**(A)** Headache and **(B)** Neck pain over time.

The results for the quantitative sensory tests, clinical examination, and self-reported outcomes over time for the three PTSS groups are presented in Table 3, Figures 1–**4** and in the following sections.

**Table 3:** Differences in outcomes for the three PTSS groups at each time point.

**Measure**	**Time**	**Subclinical (A)**		**Mild (B)**		**Clinical (C)**		**Dif. (B-A)**		**Dif. (C-A)**	
		***Est*.**	***SE***	***Est*.**	***SE***	***Est*.**	***SE***	***Est*.**	***SE***	***Est*.**	***SE***
**Pain sensitivity**											
PPDT	T0	191.2	4.9	173.5	5.6	140.7	9.7	−17.7^*^	7.4	−50.5^***^	10.9
	T1	227.8	6.1	201.3	6.4	175.5	10.7	−26.5^**^	8.8	−52.3^***^	12.3
	T2	243.6	6.6	209.6	6.8	202.1	11.9	−34.0^***^	9.5	−41.5^**^	13.6
	T4	270.4	6.9	222.6	7.1	209.2	12.4	−47.8^***^	9.9	−61.1^***^	14.2
PPT	T0	424.9	11.1	385.2	12.5	288.3	21.8	−39.7^*^	16.7	−136.6^***^	24.5
	T1	511.1	13.4	439.5	14.2	343.1	23.9	−71.6^***^	19.5	−168.0^***^	27.4
	T2	534.0	14.6	469.2	15.0	390.2	26.1	−64.8^**^	20.9	−143.8^***^	29.9
	T4	590.9	15.2	517.1	15.5	439.3	27.1	−73.9^***^	21.7	−151.6^***^	31.0
**Neck mobility**											
CROM	T0	286.0	3.6	269.7	4.1	225.8	7.1	−16.3^**^	5.4	−60.2^***^	8.0
	T1	303.3	4.4	288.1	4.7	253.2	7.8	−15.2^*^	6.4	−50.1^***^	8.9
	T2	341.4	4.8	332.1	4.9	325.2	8.6	−9.4	6.8	−16.2	9.8
	T4	339.0	5.0	329.5	5.1	300.5	8.9	−9.4	7.1	−38.5^***^	10.2
**Pain distribution**											
Palpation	T0	12.4	0.5	14.8	0.6	17.8	1.0	2.3^**^	0.8	5.4^***^	1.1
	T1	8.0	0.6	9.6	0.7	13.0	1.1	1.5	0.9	5.0^***^	1.3
	T2	4.4	0.7	5.7	0.7	7.9	1.2	1.3	1.0	3.4^*^	1.4
	T4	5.5	0.7	6.2	0.7	11.1	1.3	0.7	1.0	5.7^***^	1.5
Pain map	T0	4.4	0.2	5.3	0.2	7.0	0.4	0.9^**^	0.3	2.5^***^	0.4
	T2	3.3	0.2	4.6	0.2	5.3	0.4	1.2^***^	0.3	2.0^***^	0.5
	T4	3.9	0.4	5.1	0.4	7.5	0.9	1.1	0.6	3.6^***^	1.0
**Pain Intensity**											
Headache	T0	3.4	0.2	3.9	0.2	5.4	0.3	0.5^*^	0.2	2.0^***^	0.3
	T2	2.7	0.2	3.4	0.2	4.3	0.4	0.7^**^	0.3	1.6^***^	0.4
	T3	2.7	0.2	3.3	0.2	4.8	0.4	0.6^*^	0.3	2.1^***^	0.4
	T4	2.5	0.2	3.4	0.2	5.2	0.4	0.9^***^	0.3	2.7^***^	0.4
Neck pain	T0	3.9	0.1	4.5	0.2	5.7	0.3	0.6^**^	0.2	1.9^***^	0.3
	T2	2.5	0.2	3.3	0.2	4.4	0.3	0.8^***^	0.2	1.9^***^	0.3
	T3	2.3	0.2	3.3	0.2	4.4	0.3	1.0^***^	0.2	2.1^***^	0.3
	T4	2.3	0.2	3.3	0.2	4.8	0.3	0.9^***^	0.2	2.5^***^	0.3

*PTSS groups = Impact of event scale (IES) scores: subclinical (0–8), mild (9–25), and clinical (≥ 26)*.

*T0 = baseline within 10-day post-injury, T1–T4 = 1-, 3-, 6-, and 12-month post-injury. PPDT, pressure pain detection threshold; PPT, pressure pain tolerance; CROM, cervical range of motion; Palpation, number of painful sites with palpation; Pain map, number of body areas with pain on McGill's pain map; Headache, average level of headache on an 11-point box scale; Neck pain, average level of neck pain on an 11-point box scale; Est., Estimate based on linear mixed-effects models (LMM); SE, standard error; Dif. (B–A), Difference in scores between the mild and the subclinical PTSS groups; Dif. (C–A), Difference in scores between the clinical and the subclinical PTSS groups*.

* *p < 0.05*,

** *p ≤ 0.01*,

*** *p < 0.001*.

#### Pain Sensitivity Measures Using QSTs

The overall test of no interaction between the PTSS groups and time for the PPDTs was the rejected Wald test: χ^2^(6) = 14.41, *p* = 0.025, Figure 1A). The main departure was observed in the mild PTSS group at 12-month follow-up. The subclinical group experienced a significant steady increase over time. Moreover, both the mild and the clinical PTSS groups had significantly lower levels of PPDT at all time points compared to the subclinical PTSS group (Table 3).

The overall test of no interaction between the PTSS groups and time for PPT could not be rejected (Wald test: χ^2^(6) = 6.25, *p* = 0.396, Figure 1B), indicating no time by group interaction. Both the mild and the clinical PTSS groups had significantly lower levels of PPT at all time points compared to the subclinical PTSS group (Table 3).

#### Neck Mobility

The overall test of no interaction between the PTSS groups and time for neck mobility (CROM) was rejected (Wald test: χ^2^(6) = 29.34, *p* < 0.001, Figure 2). The main departure was observed in the clinical group, which decreased in neck mobility at 12-month follow-up, while the other groups increased from baseline to 3-month follow-up, after which they remained stable. The clinical PTSS group had significantly lower neck mobility at all time points except for 3-month follow-up compared to the subclinical group. The mild PTSS group had significantly lower neck mobility at 1- and 3-month follow-up compared to the subclinical PTSS group (Table 3).

#### Pain Distribution

The overall test of no interaction between the PTSS groups and time for palpation could not be rejected (Wald test: χ^2^(6) = 6.77, *p* = 0.343, Figure 3A), indicating no time by group interaction. The clinical PTSS group experienced significantly more painful body areas during palpation at all time points compared to the subclinical group. The mild PTSS group only experienced more painful areas at baseline compared to the subclinical PTSS group (Table 3).

The overall test of no interaction between the PTSS groups and time for the pain map could not be rejected (Wald test: χ^2^(6) = 5.77, *p* = 0.217, Figure 3B), indicating no time by group interaction. The clinical PTSS group reported significantly more painful body areas on the pain map at all time points compared to the subclinical group. The mild PTSS group only reported more painful areas on the pain map at baseline and 3-month follow-up compared to the subclinical PTSS group (Table 3).

#### Pain Intensity

The overall test of no interaction between the PTSS groups and time for headache could not be rejected (Wald test: χ^2^(6) = 10.61, *p* = 0.101, Figure 4A), indicating no time by group interaction. Both the clinical and the mild PTSS group reported significantly higher intensity of headache at all time points compared to the subclinical group (Table 3).

The overall test of no interaction between the PTSS groups and time for neck pain could not be rejected (Wald test: χ^2^(6) = 5.25, *p* = 0.512, Figure 4B), indicating no time by group interaction. Both the clinical and the mild PTSS groups reported significantly higher intensity of neck pain at all time points compared to the subclinical group (Table 3).

## Discussion

This study is, to our knowledge, the first longitudinal study to assess the potential impact of PTSS on pain sensitization with QST from within days after whiplash injury over the course of the first year. Confirming the first hypothesis, it was found that the clinical PTSS group showed increased pain sensitivity to all QSTs at all time points compared to the subclinical PTSS group. The second hypothesis was also confirmed with the clinical PTSS group, showing significantly lower neck mobility at all time points except at 3-month follow-up compared to the subclinical PTSS group. Also, the third hypothesis was confirmed with the clinical PTSS group showing more widespread pain both on clinical examination with bilateral palpation of the nine body sites and in the self-reported areas on McGill's pain map, compared to the subclinical PTSS group. Compared to the subclinical PTSS group at 12-month follow-up, the clinical PTSS group reported about two times as many painful areas in both outcomes. Also, the fourth hypothesis was confirmed with the clinical PTSS group experiencing higher levels of both headache and neck pain at all time points compared to the subclinical PTSS group.

Although not directly comparable, the present study confirms the importance of the traumatic nature of whiplash compared to non-traumatic neck pain. Previous longitudinal studies find that patients with WAD are more sensitized to pain compared to healthy controls or patients with idiopathic neck pain (14, 18). Furthermore, this study underlines the potential negative impact of posttraumatic stress reactions in the process of pain sensitization. Although the exact causal mechanisms between PTSS and pain sensitization cannot be explained by this study, the early assessment at 10 days after injury and the longitudinal approach with QST strongly indicate that traumatic stress reactions play an important role in relation to poor recovery after a whiplash injury. The clinical PTSS group not only experienced higher pain intensity and more widespread pain at 10 days after the injury but was also highly sensitized. Even at 12-month follow-up, the clinical PTSS group was below the normative range of PPDT (16, 17).

### Clinical Implications

This study emphasizes that participants with clinical levels of PTSS constitute a high-risk group that is sensitized to pain early after injury. For this reason, screening for PTSS symptoms within the 1st week after whiplash injury for those who experience high levels of pain intensity and distress may be an important clinical procedure in the assessment and treatment of WAD and, potentially, in the prevention of developing chronic WAD. This is of particular importance as conservative treatments of WAD have not shown satisfactory effects (30). Hence, early identification and treatment of posttraumatic stress reactions within days or a few weeks after the injury may be a promising approach for targeted intervention. Indeed, Sterling et al. (31) found that a preventive stress-reducing intervention in the early aftermath of whiplash injury for those with elevated levels of PTSS and pain was effective in reducing pain and disability after injury compared to treatment as usual.

## Strengths and Limitations

This study has several strengths. A longitudinal design with early assessment of PTSS and pain sensitization by using QST and clinical examinations at 10 days after the injury are major strengths. Also, the use of both QST, clinical examination, and self-report questionnaires at several follow-ups up to 12 months post-injury is unique. Finally, the sample size was relatively large, allowing several models to be assessed without compromising the statistical power. However, the study also has some limitations. Although the sample size was relatively large and all results pointed in the same direction, multiple tests were applied, which may have increased the risk of type I errors. While IES is a validated PTSD screening tool (21, 32), it only covers the two PTSD symptom clusters of avoidance and intrusion and not hyperarousal or alterations in mood and cognition, which are symptom clusters used in the ICD-11 and DSM-5 PTSD diagnoses. Hence, more up-to-date screening tools for PTSD should be applied in future studies.

## Conclusion

The present study indicates that clinical levels of PTSS early after injury (<10 days) play an important role in pain sensitization and the development of widespread pain. Participants with clinical levels of PTSS was not only highly sensitized 10 days after the injury, but also remained so for the entire follow-up of 12-month post-injury. More mechanistic and experimental studies are needed to unravel some of the cognitive, behavioral, and neurobiological mechanisms underlying the potential association between PTSS and the development of chronic WAD. Such studies need to apply up-to-date diagnostic tools for the assessment of posttraumatic stress reactions and to apply both quantitative and subjective measures of pain in a longitudinal design, preferably within hours after the injury.

## Data Availability Statement

The datasets presented in this article are not readily available because according to Danish law data cannot be shared. Requests to access the datasets should be directed to TA, tandersen@health.sdu.dk.

## Ethics Statement

The study was approved by the local Ethical Committee, The Regional Committee on Health Research Ethics of Southern Denmark. The patients/participants provided their written informed consent to participate in this study.

## Author Contributions

HK, TC, and LF designed the main study. HK participated in collecting the quantitative data. Statistical analyses were conducted by EØ. TA and HK drafted this manuscript. All authors contributed to the interpretation and discussion of the results, contributed to the design of this secondary analysis and to the formulation of the hypotheses, commented on the manuscript throughout the writing process, and approved the final version.

## Funding

This study was supported by Insurance and Pensions in Denmark, The Health Insurance Foundation, and The Council of the Danish Victims Foundation. The funders had no role in the data collection, analysis, interpretation, and reporting of results.

## Conflict of Interest

The authors declare that the research was conducted in the absence of any commercial or financial relationships that could be construed as a potential conflict of interest.

## Publisher's Note

All claims expressed in this article are solely those of the authors and do not necessarily represent those of their affiliated organizations, or those of the publisher, the editors and the reviewers. Any product that may be evaluated in this article, or claim that may be made by its manufacturer, is not guaranteed or endorsed by the publisher.
